# On the Question of Zwitterionic Intermediates in the [3+2] Cycloaddition Reactions between Aryl Azides and Ethyl Propiolate

**DOI:** 10.3390/molecules28248152

**Published:** 2023-12-18

**Authors:** Ewa Dresler, Przemysław Woliński, Aneta Wróblewska, Radomir Jasiński

**Affiliations:** 1 Łukasiewicz Research Network—Institute of Heavy Organic Synthesis “Blachownia”, Energetyków 9, 47-225 Kędzierzyn-Koźle, Poland; ewa.dresler@icso.lukasiewicz.gov.pl; 2 Institute of Organic Chemistry and Technology, Cracow University of Technology, Warszawska 24, 31-155 Cracow, Poland; przemyslaw.wolinski@pk.edu.pl; 3 Department of Organic Chemistry, Faculty of Chemistry, University of Lódź, Tamka 12, 91-403 Łódź, Poland; aneta.wroblewska@chemia.uni.lodz.pl

**Keywords:** [3+2] cycloaddition, azides, acetylenes, mechanism, Molecular Electron Density Theory

## Abstract

The molecular mechanism of the [3+2] cycloaddition reactions between aryl azides and ethyl propiolate was evaluated in the framework of the Molecular Electron Density Theory. It was found that independently of the nature of the substituent within the azide molecule, the cycloaddition process is realized via a polar but single-step mechanism. All attempts of localization as postulated earlier by Abu-Orabi and coworkers’ zwitterionic intermediates were not successful. At the same time, the formation of zwitterions with an “extended” conformation is possible on parallel reaction paths. The ELF analysis shows that the studied cycloaddition reaction leading to the 1,4-triazole proceeds by a two-stage one-step mechanism. It also revealed that both zwitterions are created by the donation of the nitrogen atom’s nonbonding electron densities to carbon atoms of ethyl propiolate.

## 1. Introduction

The chemistry of heterocyclic compounds is one of the most dynamically developing areas of science [1,2,3,4,5]. Among a number of compounds of this type, structures from the triazole group enjoy undying interest [6,7,8]. In practice, the triazole heterocyclic ring is a key molecular segment of many compounds that exhibit different types of important activities such as anti-tubercular [9,10], antiplasmodial, antimalarial [11], anticancer [12], antifungal [13,14,15], anticonvulsant [16], antiviral, antineoplastic, antihypertensive, antianxiety, and other [17]. 

The most universal methodology of the preparation of five-membered heterocycles is [3+2] cycloaddition reactions (32CA reactions) with the participation of different types of three-atom components (TACSs) [18,19,20]. The preparation of the 1,2,3-triazole molecular segment is also possible in this way due to 32CA between azides and alkynes.

**Scheme 1:**
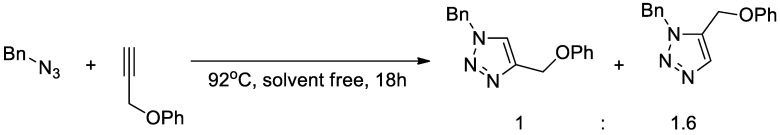
The regioselectivity of the 32CA reactions between benzyl azide and phenoxyethyne.

In general, the 32CA processes between organic azides and alkynes are realized under relatively difficult conditions and with lower regioselectivity [21] than similar processes with the participation of other nitrogen-containing TACs such as analogs of diazomethane [22], nitrile N-oxides [23,24], nitrones [25,26] nitronates [27], or azomethine ylides [28]. For example, the benzyl azide reacts with the 3-phenoxypropyne at 92 °C under solvent-free conditions yielding a mixture of both possible regioisomeric cycloadducts: 3-benzyl-4-phenoxymethyl-1,2,3-triazole and 3-benzyl-5-phenoxymethyl-1,2,3-triazole (Scheme 1) [29].

**Scheme 2:**
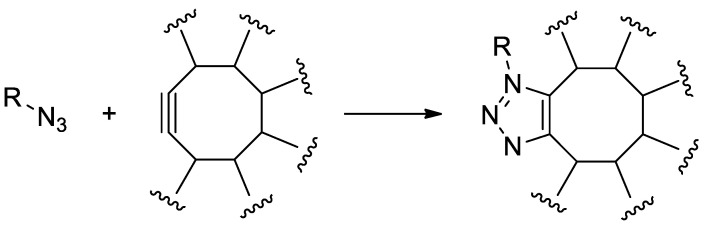
The 32CA reactions between azides and strained alkynes.

Relatively easier are analogous 32CAs with the participation of cyclooctyne analogs [30,31]. This is, however, an incidental example, determined by the specific, strained nature of the alkyne. Similar, atypical reactivity is also observed in the case of azide-fullerene C_20_ cycloadditions (Scheme 2) [32].

**Scheme 3:**
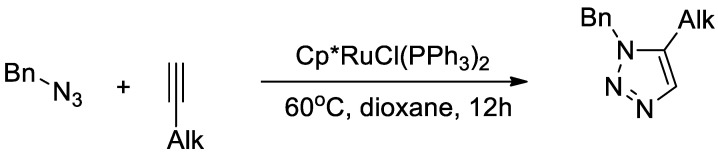
The rhutenium-catalyzed 32CA reactions between benzyl azide and simple alkynes.

Full regioselective synthesis of 1,2,3-triazoles via the 32CA reaction and under milder conditions is possible via the catalytic protocol, including the presence of cooper or rhutenium catalysts. For example, benzyl azide reacts with simple alkynes at 60 °C. The reaction is realized with full regioselectivity (Scheme 3) [33].

**Scheme 4:**
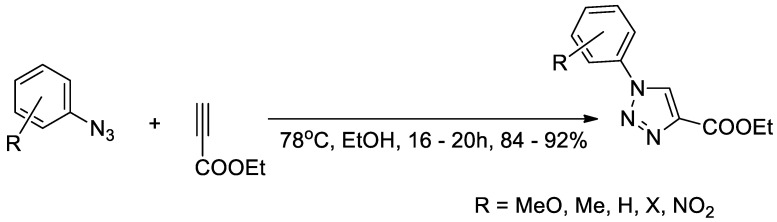
The regioselectivity and reaction conditions of the 32CAs between aryl azides **1a**–**e** and ethyl propiolate **2**.

Some years ago, Abu-Orabi and coworkers [34] described an interesting case of the 32CA reactions between aryl azides (**1a**–**e**) and ethyl propiolate (**2**) (Scheme 4). Independently of the absence of a catalyst, all analyzed reactions were realized under relatively mild conditions and (most importantly) with full regioselectivity. The authors explained this phenomenon by assuming a stepwise mechanism via a zwitterionic intermediate. The presence of the zwitterionic intermediates is, however, not confirmed in any way.

It should be underlined that, at this moment, the actual state of knowledge shed new light on the earlier interpretation of the 32CA reaction mechanism as one-step “concerted” independently of the nature of starting molecules. In particular, at this moment, several different mechanisms can be assumed within the exploration of the 32CA mechanisms; in particular, (i) polar mechanisms (the one-step mechanism, two-stage one-step mechanism, and stepwise zwitterionic mechanism) [35,36]) or (ii) non-polar mechanism (the one-step mechanism, two-stage one-step mechanism asynchronous mechanism, and stepwise biradical mechanism) [37,38]. Which of them is implemented in practice is determined by the interplay of two independent factors—the nature of electronic interactions [39] and the power of steric interactions [40]. Next, Domingo generally undermined the term “pericyclic” regarding all 32CA reactions based on the recent progress of the exploration of reaction profiles via BET and ELF techniques [41,42]. Therefore, the mechanistic aspects of the title processes evidently require deeper exploration. 

## 2. Results and Discussion

**Scheme 5:**
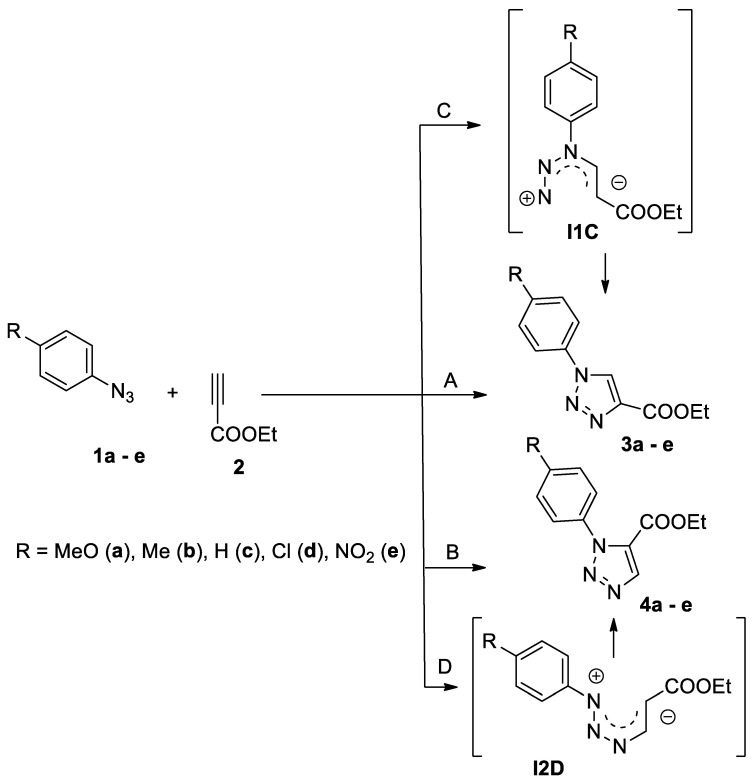
Enthalpy profiles for the reactions between cyclopentadiene **1** with isopropyl 3-nitroprop-2-enate **2** in the DCM environment in light of the wb97xd/6-311+G(d) (PCM) calculations Theoretical possible ways of the 32CA reactions between aryl azides **1a**–**e** and ethyl propiolate **2**.

The title reaction can be theoretically realized according to two competitive regioisomeric channels leading to the 3-aryl-5-carboethoxy-1,2,3-pyrazoles (**3a**–**e**) or 3-aryl-4-carboethoxy-1,2,3-pyrazoles (**4a**–**e**), respectively (Scheme 5). Within both reaction channels, however, two alternative mechanisms are possible: One-step mechanisms (paths **A** and **B**) or stepwise zwitterionic mechanisms (paths **C** and **D**).

**Figure 1:**
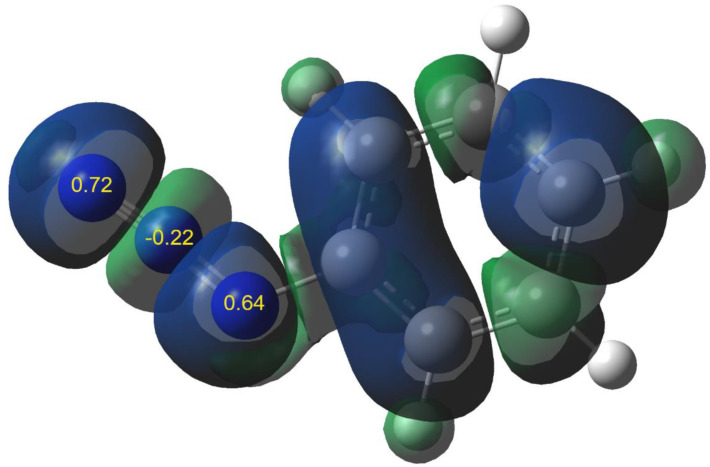
The distribution of local electrophilicities along the azide **1c** molecule.

**Figure 2:**
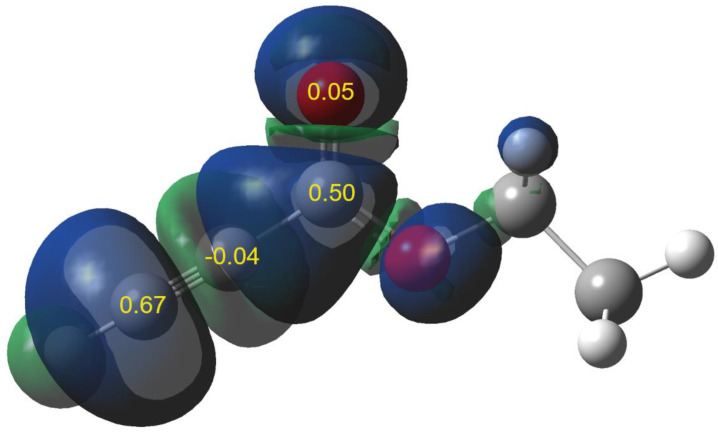
The distribution of local nucleophilicities along the ethyl propiolate **2** molecule.

In the initial step, we decided to analyze the nature of the intermolecular interactions between respective pairs of reagents (Table 1, Figure 1 and Figure 2). It was found that ethyl propiolate is characterized by global electrophilicity equal to 1.48 eV. Therefore, according to the unique Domingo electrophilicity scale [43,44], this component should be treated as a moderate electrophile. The analysis of the distribution of local electrophilicities subsequently shows that the most activated reaction center is located on the terminal carbon atom of acetylenic moiety (ω_1_ = 0.67 eV). In comparison, the local electrophilicity at the second carbon atom of the acetylenic moiety is equal to 0.04 eV. Therefore, in light of the Molecular Electron Density Theory [45], the course of the addition reaction with the participation of ethyl propiolate should be determined by the attack of nucleophilic agents on the terminal position of the ethyne moiety. On the other hand, the considered organic azides are characterized by global nucleophilicities in the range of 2.12–3.45 eV. Relatively stronger nucleophilicity properties are observed in the case of EDG-substituted azides, whereas weaker nucleophilicities are assigned to EWG-substituted azides (Table 1). It is interesting that local nucleophilicities on both important reaction centers within the NNN moiety are not significantly different and exist in the range of 0.5–0.7 eV. This suggests that in light of electronic effects, both regioisomeric cycloaddition paths should be kinetically allowed.

Within the next research step, we explored theoretically possible channels of reactions between aryl azides and ethyl propiolate, starting from the model addition **1c**+**2**. The results of the wb97xd/6-311+G(d)(PCM) calculations show that from a qualitative point of view, enthalpy profiles of reactions leading to 3-phenyl-5-carboethoxy-1,2,3-pyrazoles (**A**) or 3-phenyl-4-carboethoxy-1,2,3-pyrazoles (**B**) are very similar. In both cases, between the valley of the individual reagents and the valley of the respective cycloadduct, two critical points were detected. These are connected by the existence of the pre-reaction molecular complex (**MC**) and transition state (**TS**), respectively.

**Figure 3:**
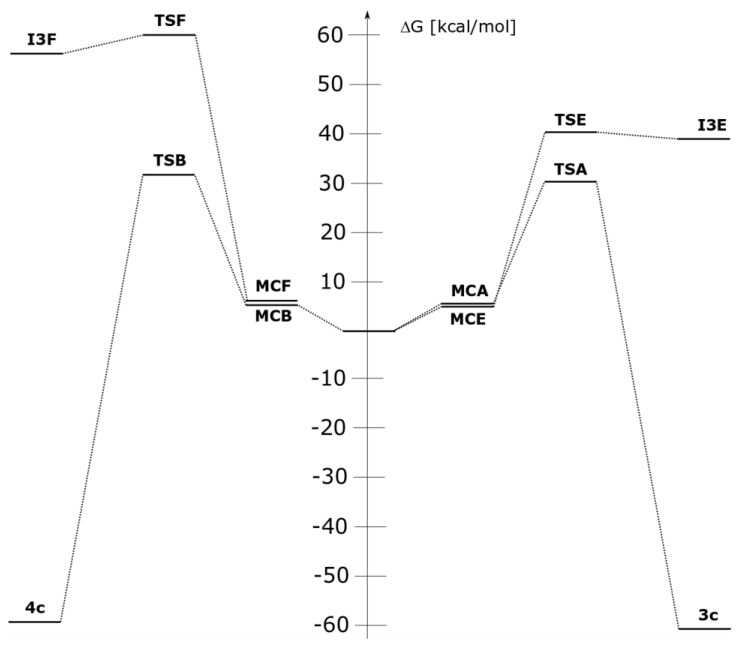
Gibbs free energy profiles for all explored paths of the reaction between phenyl azide **1c** and ethyl propiolate **2** in the ethanolic solution according to the wb97xd/6-311+G(d)(PCM) calculations.

**Figure 4:**
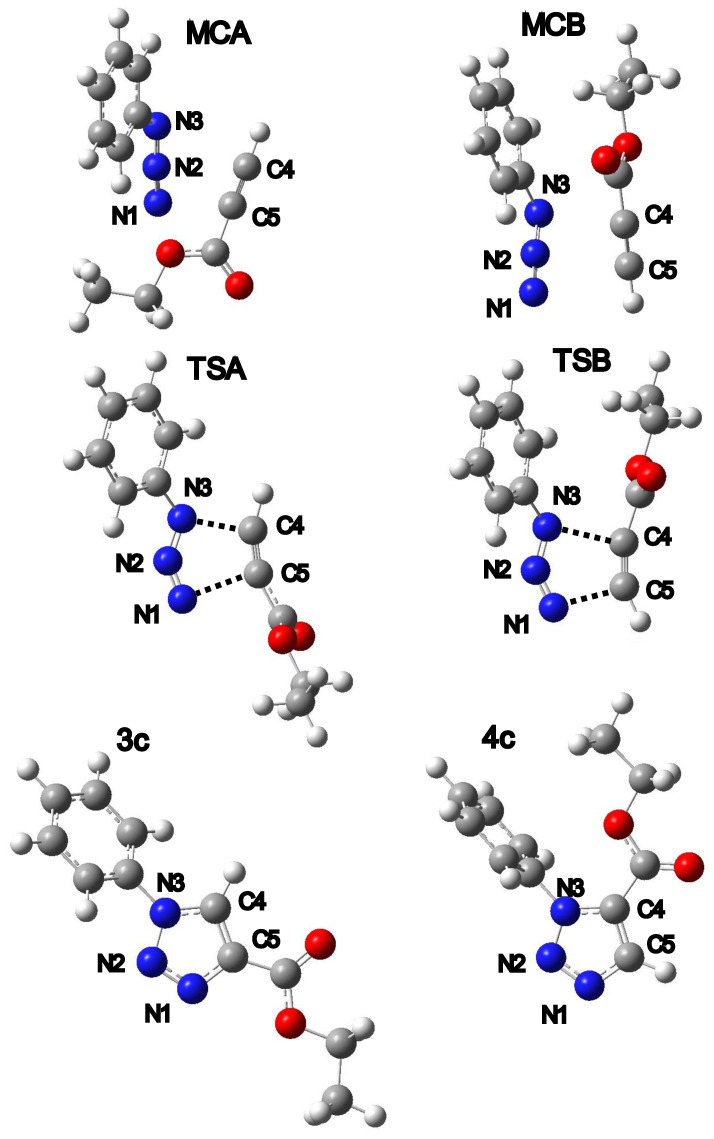
Views of critical structures of the 32CA reaction between phenyl azide **1c** and ethyl propiolate **2** in the ethanolic solution according to the wb97xd/6-311+G(d)(PCM) calculations.

Interactions between addend molecules initially lead to the formation of the pre-reaction molecular complex **MC** (**MCA** and **MCB** for paths **A** and **B**, respectively). This transformation of the reaction system is accompanied by reductions in enthalpy of 3.2 and 5.0 kcal/mol for **MCA** and **MCB**, respectively (Table 2, Figure 3). At the same time, however, a substantial reduction in the entropy of the reaction system is observed. Consequently, Gibbs free energies of the formation of MCs exhibit positive values. This excludes the possibility of the existence of MCs as thermodynamically stable intermediates. In the framework of MCs, substructures of addends adopt specific orientations, which stimulate stabilization via the coulombic interactions (Figure 4). In general, the relative orientation of the reaction centers at this stage determines the further observed regioselectivity. Therefore, localized intermediates can be interpreted as orientation complexes [46]. Within MCs, any new bonds are not formed (Table 3). Key interatomic distances of N3-C4 and C5-N1 exist beyond the area of the range typical for new C-N bonds within transition states [47,48,49]. These types of MCs were experimentally observed earlier regarding 32CAs of ozone with ethene and ethyne [50,51]. It should be underlined that both MCs do not exhibit the nature of the charge-transfer complexes [52,53]. This was confirmed by analysis of global electron density transfer (GEDT [54]) values (GEDT = 0.00e).

The further transformation of MCs on both considered reaction paths leads to TS (**TSA** and **TSB** on reaction paths **A** and **B**, respectively). Kinetic factors clearly favored the formation of the **TSA** structure, which is connected to the 3-phenyl-5-carboethoxy-1,2,3-triazole **3c**. This adduct was detected experimentally in the post-reaction mixture. It is interesting that the competitive reaction path **B** should be treated as disfavored, but not forbidden from a kinetic point of view, because the activation barrier in this case is only 1.1 kcal/mol lower than in the case of path **A**. However, one should wonder whether the authors of the work [34] carefully analyzed the post-reaction mass. The published procedure only mentions the evaporation of the solvent and recrystallization of the post-reaction mass. No detailed composition analyses were performed using, e.g., the HPLC technique [55,56,57]. We believe that the second regioisomer is likely formed there as a minority, with a yield of several percent. This is very likely because the isolated product is produced with a yield of 80–90% and the vast majority of 32CA reactions occur with a total yield close to 95–99%. This hypothesis correlates well with the above-described analysis of the local reactivity of title addends.

**Scheme 6:**
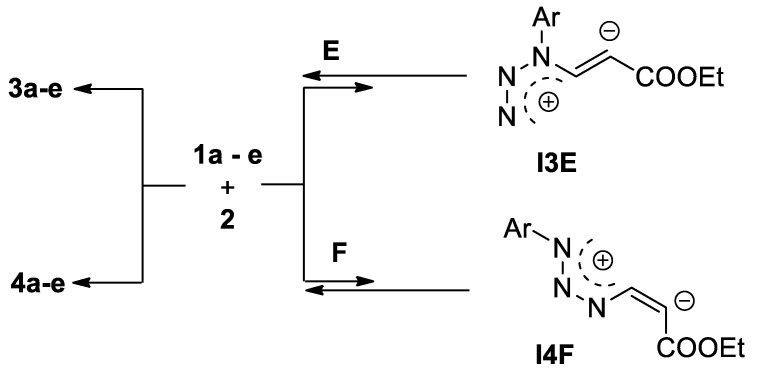
Detected ways of reactions between aryl azides **1a**–**e** and ethyl propiolate **2**.

**Figure 5:**
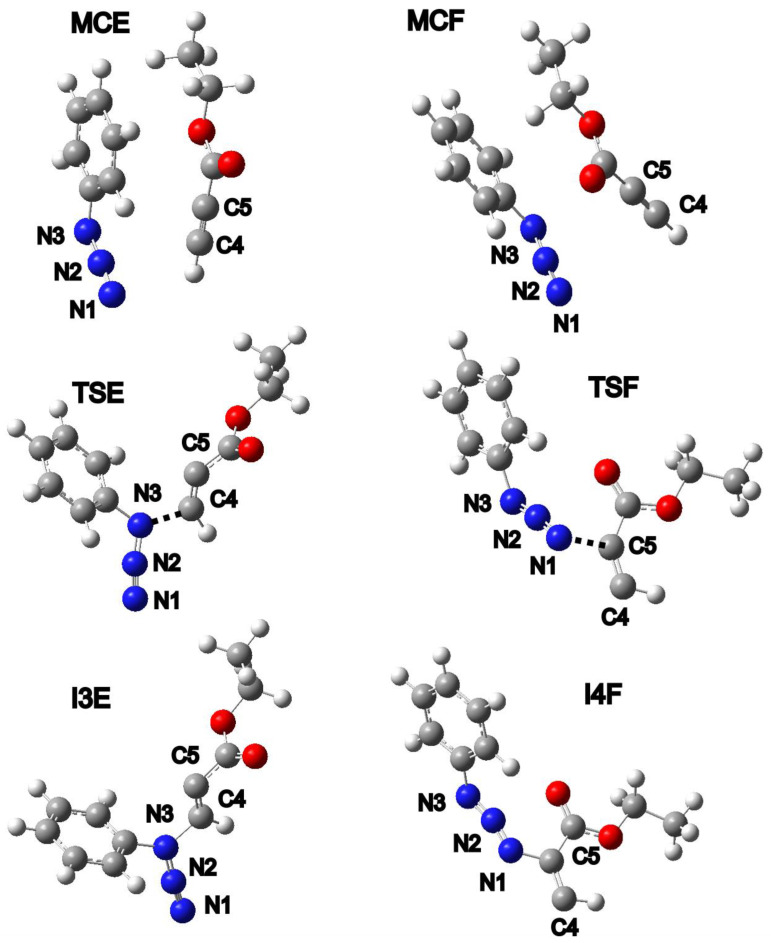
Views of critical structures of the creation of zwitterions via reaction between phenyl azide **1c** and ethyl propiolate **2** in the ethanolic solution according to the wb97xd/6-311+G(d)(PCM) calculations.

**Figure 6:**
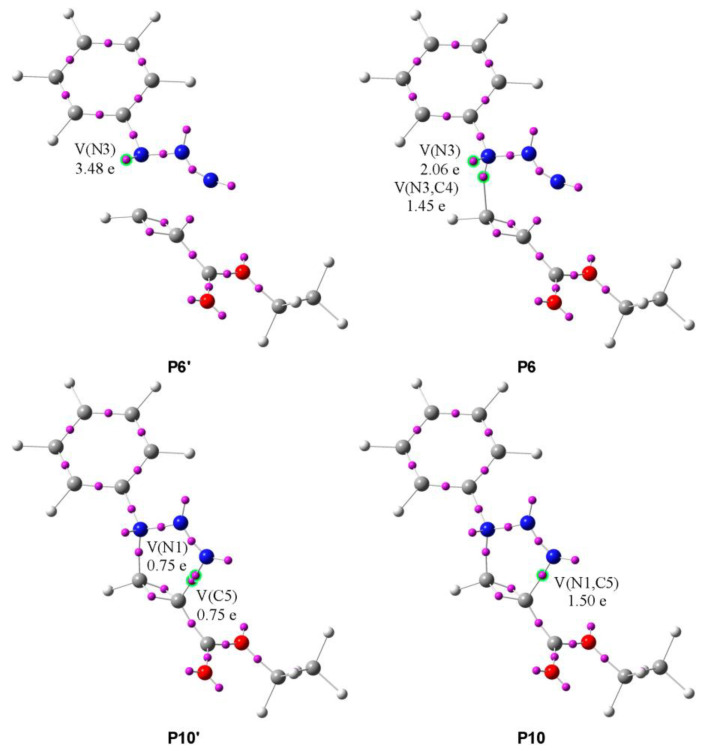
ELF valence basins attractors of structures of the most important topological changes, creation of N3-C4 and N1-C5 single bonds, during the reaction of phenyl azide **1c** and ethyl propiolate **2** along the reaction path leading to **3c**. Structures with apostrophes represent the first structure before the critical point.

Both optimized **TS**s are characterized by imaginary frequencies (−484.72 and −524.70 cm^−1^, respectively) and are structurally similar. In particular, within both TSs, key interatomic distances (N3-C4 and C5-N1) are substantially reduced in comparison to the respective MCs (Table 4). However, the kinetically favored **TSA** transition state is slightly less unsymmetrical. It should be underlined that both considered TSs exhibit evidently polar natures. This was confirmed by an examination of GEDT values (0.31 and 0.37e for **TSA** and **TSB**, respectively). The IRC analysis confirms without any doubt that optimized TSs are connected directly to the valleys of the respective MCs and respective products (Figure 3). All attempts at the localization of hypothetical zwitterionic intermediates were not successful. Therefore, postulated reaction channels **C** and **D** cannot be realized in practice. At the same time, however, we detected the possibility of the formation of other zwitterionic structures as a consequence of the interactions between azide **1c** and alkyne **2** (Scheme 6). These are not postulated intermediates **I1C** and **I2D**, but other, different adducts **I3E** and **I4F** (Figure 5 and Figure 6) characterized by an “extended” conformation [58,59]. This type of conformation excludes the possibility of the cyclization of zwitterions to cycloadducts. Their conversion to triazoles is realized via dissociation to individual reagents, and, in the next step, via one-step cycloaddition according to the **A** or **B** path. Similar dissociations are observed during the rotation around the NNCC bond.

The formation of zwitterions **I3E** and **I4F** are realized via transition states **TSE** and **TSF**, respectively. Within the considered TSs, only one new single bond is formed—N3-C4 in the case of **TSE** and N1-C5 in the case of **TSF**. It should be underlined, however, that the formation of intermediates **I3E** and **I4F** is not favored from a thermodynamic point of view, because the Gibbs free energies of the formation of **I3E** and **I4F** are substantially positive. Next, from the kinetic point of view, both these transformations should be considered forbidden due to the very high activation barriers in comparison to reaction paths **A** and **B**.

In a similar way, we examined similar reactions with the participation of azides substituted by different types of EDG or EWG groups. In all cases, we detected analogous molecular mechanisms as in the case of the addition **1c**+**2**. Therefore, the proposed mechanism can be assumed to be general for a defined group of 32CA reactions.

**Scheme 7:**
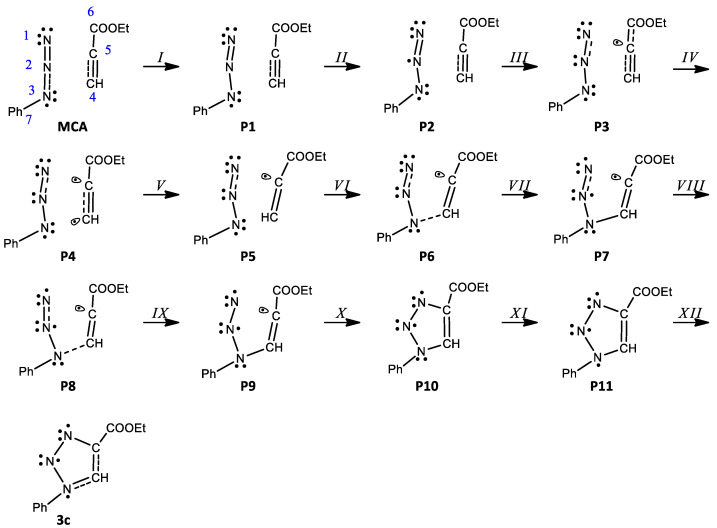
Simplified representation of the molecular mechanism of 32CA of phenyl azide **1c** and ethyl propiolate **2** by Lewis-like structures based on the topological analysis of the ELF along the reaction path leading to **3c**.

In the last step, we decided to characterize the mechanism of 32CA of phenylazide **1c** with ethyl propiolate **2** as a representative model with the bonding evolution theory (BET) [60] study along the more favorable reaction path. In Table 4 and Scheme 7, detailed BET data of the critical points of the reaction are presented. The choice requirement is the change in the electronic structure, such as the creation or disappearance of a basin compared to the previous point.

The BET study of 716 points along the reaction path revealed twelve phases following intrinsic reaction coordinates. The most important observations can be summed up as follows:

(1) In phases *I*–*II*, disynaptic basins V(N1,N2) and V′(N1,N2) go through topological changes leading to the creation of a new monosynaptic basin V(N2). First, in **P1**, disynaptic basin V′(N1,N2) disappears, transferring its electron density into V(N1,N2) and integrating 4.20e. Next, in **P2**, monosynaptic basin V(N2) representing nonbonding electron density is created with a population of 0.62e originating from V(N1,N2). The energy increases by 9.4 kcal/mol.
(2) In phases *III*–*VI*, events leading to the creation of the first new single bond can be observed. During phase *III*, the integration of disynaptic basins V(N1,N2) and V(N1,N2) decreases by 0.81e and 0.42e, respectively, increasing the population of monosynaptic basin V(N2). In **P3**, a *pseudoradical* center is created on C5 represented by the V(C5) monosynaptic basin, integrating 0.14e. An increase of 9.9 kcal/mol in energy can be observed, and the GEDT is −0.08e. Next, a short-phase *V* starts with the creation of a *pseudoradical* center on C4 integrating 0.05e; at the same time, an increase in the population in basins V(C5) and V(N2) can be seen. In **P5**, monosynaptic basin V(C4) disappears, with its electron density being transferred to the monosynaptic basin V(N3) and integrating 3.48e.
(3) Phase *VII*, d(N1-C5) = 2.104 Å and d(N3-C4) = 1.878 Å, starts with the creation of the first new C-N bond by donation of the nonbonding electron density of N3 to C4. The disynaptic basin V(N3,C4) is created with an initial population of 1.45e, while the V(N3) monosynaptic basin’s integration decreases to 2.06e, as shown by structures **P6′** and **P6** in Figure 6.
(4) In **P7**, a new monosynaptic basin V′(N1) integrating 0.38e is created with its population originating from V(N1) now integrating 3.45e. Meanwhile, a transfer of population from disynaptic basins V(C4,C5) and V′(C4,C5) of the C4-C5 double bond to V(N3,C4) and V(C5) can be observed. The GEDT is 0.14e, and the energy decreases by 15.3 kcal/mol.
(5) Phase *IX* starts with the creation of a monosynaptic basin V′(N3), integrating 0.50e, and at the same time the population of the disynaptic basin V(N3,C4) decreases to 1.33e. Next, at **P9**, basin V′(N3) disappears transferring its population back to V(N3,C4), integrating now 2.02e. Additionally, an increase in population of the monosynaptic basin V′(N1) of 0.30e originating from V(N1) can be seen. The energy decreases by 20.2 kcal/mol and the GEDT increases to 0.24e.
(6) The second new C-N bond is created at **P10**, d(N1-C5) = 1.720 Å and d(N3-C4) = 1.561 Å, by shearing of the *pseudoradical* center C5 and nonbonding electron density of N1. The monosynaptic basin V(C5) and V′(N1), both integrating 0.75e, merge to create the new disynaptic basin V(N1,C5) with a population of 1.50e, as shown by structures **P10′** and **P10** in Figure 6.
(7) Phase *XII* starts with the disappearance of V′(C4,C5) with the transfer of its population to V(C4,C5), now integrating 3.67e. A decrease in energy of 27.3 kcal/mol and a GEDT of 0.29e can be observed.
(8) The 32CA of phenylazide **1c** with alkyne **2** takes place via a *two-stage one-step* mechanism of the attack of nucleophilic N3 of the azide on the most electrophilic carbon C4 of the alkyne.

**Scheme 8:**
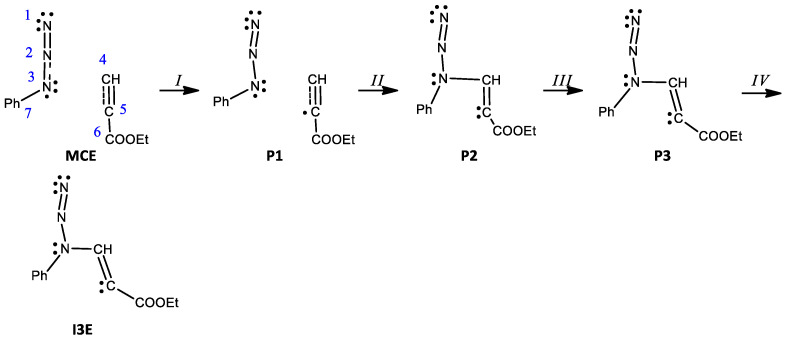
Simplified representation of the molecular mechanism of the reaction of phenyl azide **1c** and ethyl propiolate **2** by Lewis-like structures based on the topological analysis of ELF along the reaction path leading to **I3E**.

**Figure 7:**
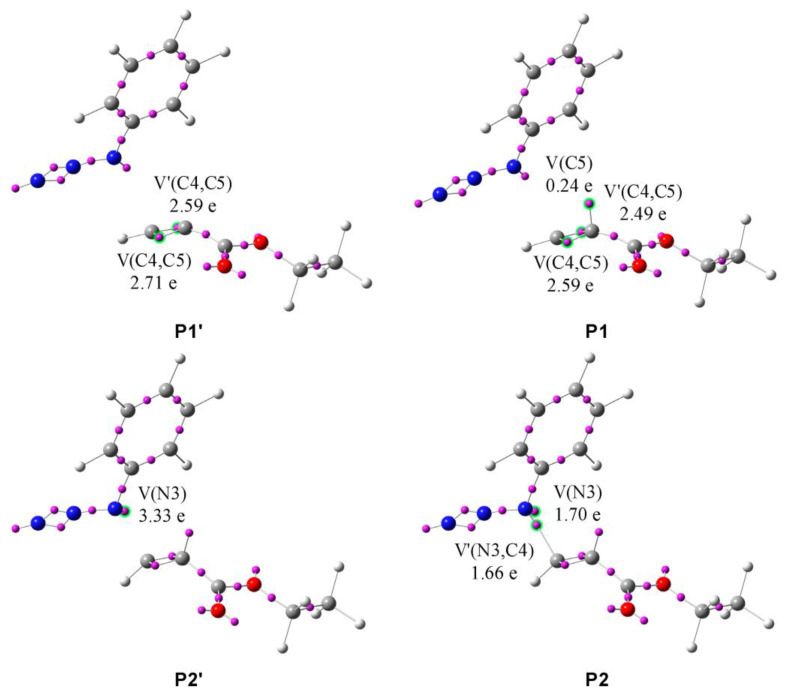
ELF valence basins attractors of structures of the most important topological changes, creation of negative charge on C5 and creation of N3-C4 single bond, during the reaction of phenyl azide **1c** and ethyl propiolate **2** along the reaction path leading to **I3E**. Structures with apostrophes represent the first structure before the critical point.

In the same way, the process of N3-C4 zwitterion creation has been evaluated (Table 5, Scheme 8). Analysis of hte 264 points along the IRC revealed four phases, of which the most important aspects are:

(1) In **P1**, a monosynaptic basin V(C5) integrating 0.24e is created with its population originating from two disynaptic basins V(C4,C5) and V′(C4,C5), as shown by **P1′** and **P1** in Figure 7. The energy increases by 17.0 kcal/mol and the GEDT is −0.03e.
(2) At **P2**, the most significant topological change can be observed, and a new bond is created by sharing the N3 nonbonding electron density with C4. The monosynaptic basin V(N3) is depopulated and a new disynaptic basin V(N3,C4) is created, integrating 1.68e, as shown by structures **P2′** and **P2** in Figure 7. The GEDT increases to −0.31e, and another significant rise in energy of 17.7 kcal/mol can be seen.
(3) The last phase IV starts with the creation of monosynaptic basin V′(N3) integrating 0.69e. The energy decreases by 0.9 kcal/mol, while the GEDT increases to −0.51e. The energy of the final structure of the zwitterion **I3E** is 0.5 kcal/mol lower and the GEDT reaches −0.53e.

**Table 5:** ELF valence basin populations of the IRC points, **MCE**—**I3E**, defining the four different phases characterizing the reaction of the phenyl azide **1c** and ethyl propiolate **2**. The stationary points **1c**, **2**, **MCE**, **TSE**, and **I3E** are also included. Distances are given in angstroms, Å, electron populations in the average number of electrons, [e], relative energies in kcal·mol^−1^, and GEDT values in an average number of electrons, [e].

Structures	1c	2	MCE		P1		P2	TSE	P3		I3E
Phases				*I*		*II*		*III*		*IV*	
d1(N3-C4)			3.460		2.474		1.798	1.710	1.532		1.518
GEDT			0.00		−0.03		−0.31	−0.38	−0.51		−0.53
dE			−6.2		10.8		28.5	28.8	27.6		27.1
V(N1)	3.76		3.77		3.80		3.67	3.64	3.59		3.59
V(N1.N2)	2.34		2.32		2.50		2.67	2.65	2.37		2.32
V′(N1.N2)	1.80		1.81		1.67		1.70	1.75	2.02		2.06
V(N2.N3)	2.50		2.51		2.47		2.28	2.25	2.27		2.28
V(N3)	3.37		3.37		3.28		1.70	1.54	1.25		1.10
V(N3.C7)	1.83		1.84		1.88		1.81	1.80	1.80		1.79
V(C4.C5)		2.56	2.56		2.49		2.06	2.00	1.83		1.81
V′(C4.C5)		2.73	2.72		2.59		2.10	2.04	1.88		1.85
V(C5.C6)		2.40	2.40		2.41		2.31	2.32	2.32		2.29
V′(N3)									0.69		0.84
V(N3.C4)							1.68	1.90	1.59		1.61
V(C4)					0.24		1.35	1.47	1.81		1.90

**Scheme 9:**
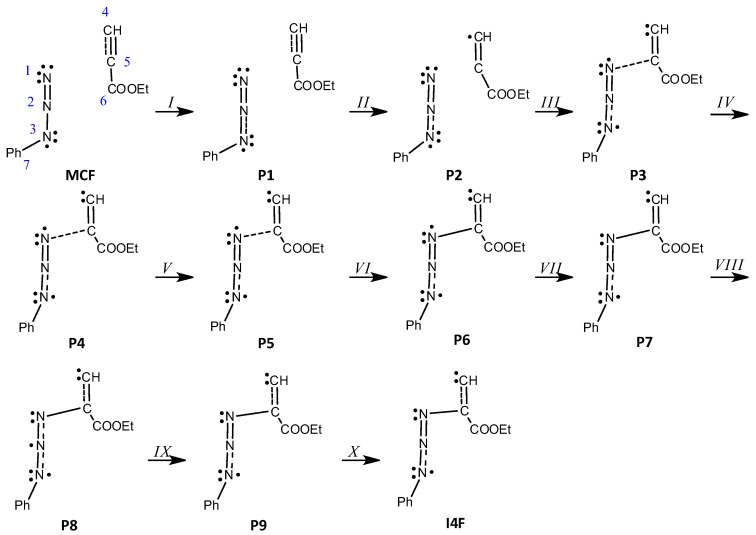
Simplified representation of the molecular mechanism of the reaction of phenyl azide **1c** and ethyl propiolate **2** by Lewis-like structures based on the topological analysis of the ELF along the reaction path leading to **I4F**.

**Figure 8:**
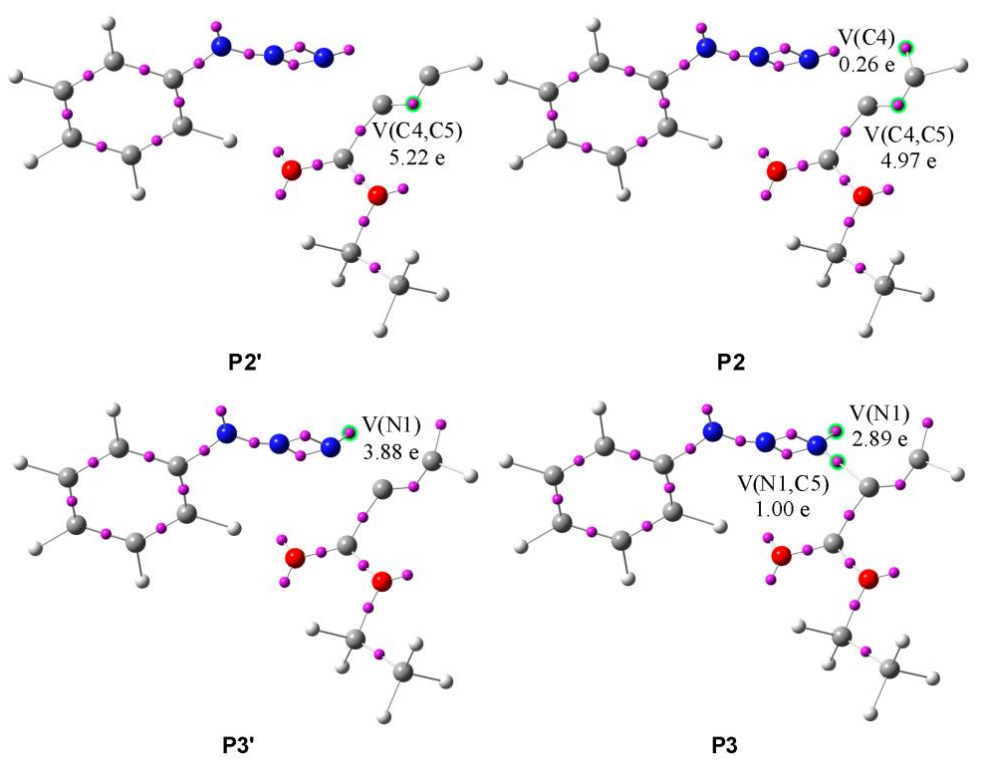
ELF valence basins attractors of structures of the most important topological changes, creation of negative charge on C4, and creation of N1-C5 single bond during the reaction of phenyl azide **1c** and ethyl propiolate **2** along the reaction path leading to **I4F**. Structures with apostrophes represent the first structure before the critical point.

The analysis of the mechanism of formation of less favorable zwitterion N1-C5 (Table 6, Scheme 9) revealed ten phases during which the most important topological changes are:

(1) The first topological change takes place in **P1** where disynaptic basin V′(C4,C5) merges with V(C4,C5), now integrating 5.19e, and an increase in energy of 27.8 kcal/mol can be observed. Next, phase *III* starts with the creation of a monosynaptic basin V(C4), with an initial population of 0.26e, which represents the developing negative charge of the zwitterion, as shown in Figure 8 by **P2′** and **P2**. The energy increases by 8.0 kcal/mol and the GEDT is −0.06e.
(2) With the start of phase *IV*, another mayor jump in energy can be seen; it rises by 17.4 kcal/mol. The new bond between C5 and N1 is created by shearing the nonbonding electron density of N1 with C5. The disynaptic basin V(N1,C5) is created with the initial population of 1.00e coming from the monosynaptic basin V(N1), as shown in Figure 8 by **P3′** and **P3**. The GEDT increases to −0.26e.
(3) In phases *V*-*X*, further reorganization of the electron density can be observed. The disynaptic basin V(C4,C5) and monosynaptic basins V(N1) and V(N3) gradually depopulate while the disynaptic basins V(N1,C5), V(N2,N3), and V′(N2,N3) and monosynaptic basin V(C4) increase in population. The final structure of the zwitterion **I4F** possesses 5.1 kcal/mol lower energy than the transition state.

**Table 6:** ELF valence basin populations of the IRC points, **MCF**—**I4F**, defining the ten different phases characterizing the reaction of the phenyl azide **1c** and ethyl propiolate **2**. The stationary points **1c**, **2**, **MCF**, **TSF**, and **I4F** are also included. Distances are given in angstroms, Å, electron populations in the average number of electrons, [e], relative energies in kcal·mol-1, and GEDT values in an average number of electrons, [e].

Structures	1c	2	MCF		P1		P2		P3		P4		P5	TSF	P6		P7		P8		P9		I4F
Phases				*I*		*II*		*III*		*IV*		*V*		*VI*		*VII*		*VIII*		*IX*		*X*	
d1(N1-C5)			3.740		2.189		2.075		1.817		1.778		1.722	1.700	1.558		1.492		1.446		1.443		1.428
GEDT			0.00		−0.03		−0.06		−0.26		−0.30		−0.36	−0.38	−0.49		−0.51		−0.50		−0.50		−0.48
dE			−6.6		21.2		29.2		46.6		47.7		48.4	48.5	47.1		46.1		44.8		44.5		43.4
V(N1)	3.76		3.74		3.74		3.74		2.89		2.78		2.68	2.65	2.53		2.49		2.46		2.45		2.41
V(N1.N2)	1.80		1.81		1.90		1.90		1.79		1.77		1.74	1.73	1.70		3.71		2.90		3.79		3.84
V′(N1.N2)	2.34		2.37		2.24		2.23		2.10		2.08		2.05	2.04	2.01								
V(N2.N3)	2.50		2.47		2.54		2.57		2.80		2.84		2.90	2.92	1.53		1.50		1.49		1.51		2.85
V(N3)	3.37		3.39		3.34		3.29		3.07		3.03		2.98	2.95	2.83		2.79		2.78		2.78		2.82
V(N3.C7)	1.83		1.83		1.84		1.86		1.89		1.90		1.91	1.91	1.94		1.95		1.97		1.97		1.95
V(C4.C5)		2.73	2.78		5.19		4.97		4.07		1.95		3.90	3.86	3.63		3.53		3.42		3.41		3.37
V′(C4.C5)		2.56	2.56								2.06												
V(C5.C6)		2.40	2.41		2.47		2.49		2.46		2.43		2.41	2.40	2.37		2.36		2.36		2.36		2.36
V(N1.C5)									1.00		1.17		1.35	1.40	1.68		1.80		1.92		1.93		2.01
V(N2)																			0.90				
V′(N2.N3)															1.47		1.49		1.41		1.39		1.39
V(C4)							0.26		1.62		1.73		1.87	1.91	2.11		2.16		2.19		2.19		2.20

## 3. Computational Details

All calculations reported in this paper were performed using the “Ares” infrastructure in the “Cyfronet” computational center in Cracow. Hybrid functional wb97xd with the 6-311+G(d) basis set included in the GAUSSIAN 09 package [61] was used. Previously, we found that the wb97xd/6-311+G(d) calculations illustrate the molecular mechanisms of several different 32CA reactions well [25,49,62,63].

Global electronic properties of reactants were estimated according to the equations recommended by *Parr* and *Domingo* [64,65,66]. According to *Domingo’s* recommendation, for this purpose, the B3LYP/6-31G(d) level of theory was used. In particular, the electronic chemical potentials (μ) and chemical hardness (η) were evaluated in terms of one-electron energies of FMO (E_HOMO_ and E_LUMO_) using Equations (1) and (2):

μ ≈ (E_HOMO_ + E_LUMO_)/2 (1)

η ≈ E_LUMO_ − E_HOMO_ (2)

Next, the values of μ and η were then used for the calculation of global electrophilicity (ω) according to the Formula (3) [66], and the global nucleophilicity (N) [67] can be expressed in terms of Equation (4)

ω = μ^2^/2η (3)

N = E_HOMO_ − E_LUMO (rerracyanoethene)_ (4)

The local electrophilicity (ω_k_) [68] condensed to atom *k* was calculated by projecting the index ω onto any reaction center *k* in the molecule using the *Parr* function P^+^_k_:

ω_k_ = P^+^_k_·ω (5)

The local nucleophilicity (N_k_) [68] condensed to atom *k* was calculated using global nucleophilicity N and *Paar* function P^−^_k_ according to the formula:

N_k_ = P^−^_k_·N (6)

For structure optimization of the reactants, intermediates, and products, the *Berny* algorithm was applied. First-order saddle points were localized using the QST2 or QST3 procedure. Stationary points were checked by vibrational frequency analyses to determine whether they constituted minima or maxima on the potential energy surface (PES). All transition structures showed a single imaginary frequency (ν_i_), whereas reactants, products, and pre-reaction complexes had none. The intrinsic reaction coordinate (IRC) path was traced in order to check the energy profiles connecting each transition structure to the two associated minima of the proposed mechanism. The calculations were carried out for the simulated presence of ethanol as the reaction medium (the PCM model [69] was used). All calculations were performed for 298 K and 1 atm pressure. The absolute entropies of critical structures (*S*_298_) were estimated from the complete vibrational analysis. Enthalpies (*H*_298_) were corrected to Gibbs free energies (*G*_298_) using the calculated entropies.

Global electron density transfer (GEDT) [54] was calculated according to Formula (7):

GEDT = −Σq_A_ (7)

where q_A_ is the net charge and the sum is taken for all the atoms of nitroalkene. 

Indexes of σ-bonds development (l) were calculated according to Formula (8) [36]:

(8) $$l_{X-Y}=1-\frac{r_{X-Y}^{\mathrm{TS}}-r_{X-Y}^{P}}{r_{X-Y}^{P}}$$

where r^TS^_X−Y_ is the distance between the reaction centers X and Y in the transition structure and r^P^_X−Y_ is the same distance in the corresponding product.

The kinetic parameters and essential properties of critical structures are presented in Table 2 and Table 3. Consistent with the previous convention [70,71], in this paper, the letters **MC** and **TS** are designated to the pre-reaction complex and TS, respectively. They are distinguished by appending the letters **A**–**D** depending on the reaction pathway. 

The Electron Localization Function (ELF) [72] analysis was performed using the TopMod 09 package [73] at the standard cubical grid of step size of 0.1 Bohr. To visualize the molecular geometries and ELF basin attractors, the GaussView program [74] was used.

## 4. Conclusions

The wb97xd/6-311+G(d) computational study evidently underlies the hypothesis of the stepwise mechanism via a zwitterionic intermediate of the 32CA reactions between aryl azides and ethyl propiolate. Our investigations show that all title reactions are realized via a polar one-step reaction mechanism without the intervention of zwitterionic intermediates. It should be underlined, however, that the new single bond within the formed triazole ring is always formed faster at the activated electrophilically terminal carbon atom on the acetylenic moiety. Some zwitterionic intermediates can be theoretically formed in competitive reaction paths. These transformations are, however, not favored from thermodynamic and kinetic points of view. Additionally, our quantumchemical calculations suggest that within the reaction course, a minor amount of 3-aryl-4-carboethoxy-1,2,3-triazole not isolated and not identified by Abu-Orabi and co-workers can be formed. This suggests a further, deeper experimental reexamination of title reactions should be performed. This will be the subject of our future research. The ELF analysis revealed that the 32CA reaction takes place via a non-concerted two-stage one-step mechanism during which the first single bond is created by donation of the nonbonding electron density of N3 to C4, and the second single bond is created by shearing of the *pseudoradical* center C5 with the nonbonding electron density of N1. Meanwhile, new bonds in the theoretically possible zwitterions are created by the donation of the nonbonding electron density of the appropriate nitrogen atom to the carbon atom.

## Figures and Tables

**Table 1:** Global and local nucleophilicities at azide molecules **1a**–**c** (in eV).

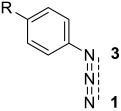				
No	R	N	N_1_	N_3_
**1a**	MeO	3.45	0.65	0.47
**1b**	Me	3.11	0.71	0.57
**1c**	H	2.92	0.72	0.64
**1d**	Cl	2.80	0.60	0.51
**1e**	NO_2_	2.12	0.53	0.52

**Table 2:** Energetic characterization of key transitions within 32CA reaction between aryl azides **1a**–**e** and ethyl propiolate **2** in the ethanolic solution according to the wb97xd/6-311+G(d)(PCM) calculations (ΔH, ΔG are in kcal/mol; ΔS are in cal/molK).

Reaction	Path	Transition	ΔH	ΔS	ΔG
**1a+2**	**A**	**1a+2→MCA**	−3.3	−25.5	4.3
		**1a+2→TSA**	17.4	−40.7	29.6
		**1a+2→3a**	−76.6	−48.6	−62.1
	**B**	**1a+2→MCB**	−3.7	−39.3	8.0
		**1a+2→TSB**	17.1	−44.3	30.3
		**1a+2→4a**	−74.4	−45.4	−60.9
	**E**	**1a+2→MCE**	−5.9	−37.3	5.2
		**1a+2→TSE**	28.3	−35.3	38.8
		**1a+2→I3E**	27.2	−39.0	38.8
	**F**	**1a+2→MCF**	−5.7	−35.3	4.9
		**1a+2→TSF**	46.8	−41.7	59.3
		**1a+2→I4F**	42.6	−43.6	55.6
**1b+2**	**A**	**1b+2→MCA**	−5.7	−34.1	4.4
		**1b+2→TSA**	17.8	−42.4	30.4
		**1b+2→3b**	−76.1	−49.2	−61.5
	**B**	**1b+2→MCB**	−6.0	−39.1	5.7
		**1b+2→TSB**	17.0	−49.2	31.7
		**1b+2→4b**	−74.3	−53.5	−58.4
	**E**	**1b+2→MCE**	−6.0	−39.1	5.7
		**1b+2→TSE**	28.3	−43.9	41.3
		**1b+2→I3E**	28.1	−36.8	39.0
	**F**	**1b+2→MCF**	−5.7	−34.1	4.4
		**1b+2→TSF**	47.6	−39.7	59.5
		**1b+2→I4F**	43.8	−41.6	56.2
**1c+2**	**A**	**1c+2→MCA**	−3.2	−29.0	5.4
		**1c+2→TSA**	18.0	−41.0	30.2
		**1c+2→3c**	−75.7	−48.6	−61.3
	**B**	**1c+2→MCB**	−5.0	−32.6	4.7
		**1c+2→TSB**	18.0	−44.8	31.3
		**1c+2→4c**	−73.2	−45.1	−59.7
	**E**	**1c+2→MCE**	−4.9	−34.4	5.3
		**1c+2→TSE**	29.4	−37.5	40.5
		**1c+2→I3E**	28.7	−35.9	39.4
	**F**	**1c+2→MCF**	−5.3	−34.7	5.0
		**1c+2→TSF**	48.3	−40.5	60.4
		**1c+2→I4F**	44.6	−42.8	57.4
**1d+2**	**A**	**1d+2→MCA**	−3.4	−30.4	5.7
		**1d+2→TSA**	18.2	−41.7	30.6
		**1d+2→3d**	−75.3	−48.7	−60.8
	**B**	**1d+2→MCB**	−5.9	−36.6	5.0
		**1d+2→TSB**	18.1	−44.1	31.2
		**1d+2→4d**	−73.1	−47.1	−59.0
	**E**	**1d+2→MCE**	−5.9	−36.3	4.9
		**1d+2→TSE**	29.8	−37.4	41.0
		**1d+2→I3E**	29.4	−38.0	40.7
	**F**	**1d+2→MCF**	−5.6	−34.5	4.7
		**1d+2→TSF**	48.8	−41.3	61.1
		**1d+2→I4F**	45.3	−41.7	57.7
**1e+2**	**A**	**1e+2→MCA**	−4.2	−35.4	6.3
		**1e+2→TSA**	18.7	−38.9	30.2
		**1e+2→3e**	−73.5	−48.7	−58.9
	**B**	**1e+2→MCB**	−5.2	−35.0	5.2
		**1e+2→TSB**	18.9	−42.6	31.6
		**1e+2→4e**	−71.0	−46.7	−57.1
	**E**	**1e+2→MCE**	−5.6	−36.8	5.4
		**1e+2→TSE**	31.9	−38.9	43.4
		**1e+2→I3E**	32.0	−33.9	42.1
	**F**	**1e+2→MCF**	−5.3	−34.8	5.0
		**1e+2→TSF**	51.0	−40.9	63.2
		**1e+2→I4F**	48.0	−40.7	60.2

**Table 3:** Key parameters of critical structures of the 32CA reaction between aryl azides **1a**–**e** and ethyl propiolate **2** in the ethanolic solution according to the wb97xd/6-311+G(d)(PCM) calculations.

Reaction	Path	Structure	Interatomic Distances r [Å]					GEDT
			N1-N2	N2-N3	N3-C4	C4-C5	C5-N1	[e]
**1a+2**	**A**	**1a**	1.127	1.226				
		**2**				1.200		
		**MCA**	1.127	1.226	3.447	1.199	3.403	
		**TSA**	1.149	1.254	2.031	1.232	2.279	0.30
		**3a**	1.290	1.349	1.343	1.375	1.359	
	**B**	**MCB**	1.125	1.227	3.655	1.200	3.798	
		**TSB**	1.160	1.251	2.191	1.231	2.075	0.25
		**4a**	1.302	1.332	1.361	1.374	1.350	
	**E**	**MCE**	1.127	1.226	3.523	1.199	4.030	
		**TSE**	1.119	1.246	1.720	1.260	4.561	0.75
		**I3E**	1.114	1.257	1.515	1.303	4.548	0.89
	**F**	**MCF**	1.126	1.227	3.907	1.199	3.893	
		**TSF**	1.156	1.195	4.459	1.284	1.707	0.55
		**I4F**	1.177	1.192	4.661	1.355	1.428	0.70
**1b**+**2**	**A**	**1b**	1.126	1.227				
		**MCA**	1.126	1.227	3.461	1.199	3.416	
		**TSA**	1.149	1.253	2.033	1.232	2.272	0.31
		**3b**	1.289	1.349	1.343	1.375	1.359	
		**MCB**	1.126	1.227	3.649	1.199	3.534	
	**B**	**TSB**	1.159	1.250	2.193	1.231	2.066	0.26
		**4b**	1.302	1.332	1.361	1.374	1.349	
		**MCE**	1.126	1.227	3.491	1.199	4.047	
	**E**	**TSE**	1.119	1.246	1.717	1.261	4.547	0.74
		**I3E**	1.114	1.256	1.516	1.303	4.550	0.90
		**MCF**	1.125	1.228	3.949	1.199	4.052	
	**F**	**TSF**	1.154	1.195	4.502	1.285	1.702	0.55
		**I4F**	1.175	1.192	4.663	1.354	1.428	0.68
**1c**+**2**	**A**	**1c**	1.125	1.228				
		**MCA**	1.125	1.228	3.430	1.199	3.412	
		**TSA**	1.149	1.254	2.038	1.232	2.264	0.31
		**3c**	1.289	1.349	1.344	1.374	1.360	
		**MCB**	1.125	1.228	3.684	1.200	3.326	
**H**	**B**	**TSB**	1.159	1.251	2.195	1.231	2.061	0.27
		**4c**	1.302	1.332	1.361	1.374	1.349	
		**MCE**	1.125	1.228	3.459	1.199	4.056	
	**E**	**TSE**	1.118	1.247	1.710	1.263	4.525	0.76
		**I3E**	1.114	1.256	1.518	1.303	4.537	0.87
		**MCF**	1.125	1.229	3.886	1.199	3.740	
	**F**	**TSF**	1.153	1.196	4.538	1.286	1.700	0.55
		**I4F**	1.173	1.193	4.665	1.354	1.428	0.68
**1d**+**2**	**A**	**1d**	1.124	1.229				
		**MCA**	1.125	1.229	3.445	1.199	3.426	
		**TSA**	1.149	1.255	2.046	1.232	2.253	0.29
		**3d**	1.288	1.350	1.344	1.374	1.360	
		**MCB**	1.124	1.229	3.598	1.199	3.568	
	**B**	**TSB**	1.159	1.252	2.197	1.231	2.060	0.24
		**4d**	1.301	1.333	1.361	1.374	1.350	
		**MCE**	1.124	1.229	3.433	1.199	4.036	
	**E**	**TSE**	1.118	1.248	1.706	1.264	4.517	0.75
		**I3E**	1.114	1.257	1.521	1.302	4.526	0.89
		**MCF**	1.124	1.230	3.949	1.199	4.079	
	**F**	**TSF**	1.152	1.196	4.548	1.287	1.695	0.55
		**I4F**	1.173	1.194	4.663	1.355	1.426	0.68
**1e**+**2**	**A**	**1e**	1.122	1.234				
		**MCA**	1.122	1.234	3.419	1.200	3.464	
		**TSA**	1.149	1.258	2.073	1.231	2.217	0.27
		**3e**	1.286	1.352	1.346	1.372	1.361	
		**MCB**	1.122	1.234	3.741	1.200	3.416	
	**B**	**TSB**	1.157	1.256	2.209	1.231	2.041	0.22
		**4e**	1.299	1.335	1.362	1.373	1.351	
		**MCE**	1.121	1.235	3.388	1.199	4.099	
	**E**	**TSE**	1.116	1.252	1.690	1.268	4.454	0.75
		**I3E**	1.113	1.261	1.530	1.303	4.339	0.87
		**MCF**	1.121	1.235	3.978	1.199	4.218	
	**F**	**TSF**	1.149	1.199	4.615	1.289	1.681	0.55
		**I4F**	1.169	1.197	4.665	1.355	1.424	0.65

**Table 4:** ELF valence basin populations of the IRC points, MC **1c**–**3c**, defining the twelve different phases characterizing the reaction of the phenyl azide **1c** and ethyl propiolate **2**. The stationary points **1c**, **2**, **MCA**, **TSA**, and **3c** are also included. Distances are given in angstroms, Å, electron populations in an average number of electrons, [e], relative energies in kcal·mol^−1^, and GEDT values in an average number of electrons, [e].

Structures	1c	2	MCA		P1		P2		P3	TSA	P4		P5		P6		P7		P8		P9		P10		P11		3c
Phases				*I*		*II*		*III*		*IV*		*V*		*VI*		*VII*		*VIII*		*IX*		*X*		*XI*		*XII*	
d2(N1-C5)			3.431		3.024		2.693		2.335	2.263	2.179		2.108		2.104		1.931		1.910		1.743		1.720		1.477		1.360
d1(N3-C4)			3.137		2.717		2.433		2.105	2.037	1.953		1.883		1.878		1.718		1.701		1.575		1.561		1.435		1.344
GEDT			0.00		−0.03		−0.06		−0.08	−0.07	−0.03		0.01		0.02		0.14		0.15		0.24		0.24		0.29		0.25
dE			−4.5		0.7		6.9		16.8	17.5	16.3		13.2		12.9		−2.4		−4.8		−25.0		−28.6		−55.9		−73.1
V(N1)	3.76		3.77		3.76		3.74		3.70	3.70	3.72		3.75		3.75		3.45		3.42		3.20		3.19		3.00		2.89
V(N1.N2)	2.34		2.32		4.20		3.77		2.96	2.87	2.79		2.72		2.72		2.56		2.54		2.41		2.39		2.18		2.04
V′(N1.N2)	1.80		1.82																								
V(N2.N3)	2.50		2.51		2.46		2.31		1.89	1.83	1.77		1.74		1.73		1.70		1.69		1.70		1.70		1.72		1.71
V(N3)	3.37		3.37		3.31		3.26		3.25	3.27	3.30		3.48		2.06		1.89		1.87		1.73		1.72		1.43		0.59
V(N3.C7)	1.83		1.84		1.87		1.9		1.91	1.89	1.88		1.86		1.85		1.83		1.82		1.82		1.82		1.86		2.02
V(C4.C5)		2.56	2.52		2.48		2.38		2.43	2.39	2.31		2.20		2.19		2.04		2.02		1.93		1.92		3.67		3.36
V′(C4.C5)		2.73	2.75		2.78		2.87		2.56	2.42	2.32		2.21		2.21		2.05		2.04		1.98		1.97				
V(C5.C6)		2.4	2.40		2.43		2.46		2.51	2.45	2.42		2.4		2.4		2.35		2.35		2.31		2.3		2.25		2.38
V(N1.C5)																							1.50		1.93		2.23
V(N3.C4)															1.45		1.78		1.33		2.02		2.04		2.29		3.00
V′(N1)																	0.38		0.43		0.73						
V(N2)							0.62		1.88	2.06	2.22		2.35		2.35		2.59		2.62		2.77		2.79		3.00		3.16
V′(N3)																			0.50								
V(C4)											0.05																
V(C5)									0.14	0.36	0.49		0.58		0.59		0.71		0.72		0.75						

## Data Availability

Data are contained within the article.

## References

[1] Katritzky A.R. (2004). Introduction: Heterocycles. Chem. Rev..

[2] Kerru N., Gummidi L., Maddila S., Gangu K.K., Jonnalagadda S.B. (2020). A Review on Recent Advances in Nitrogen-Containing Molecules and Their Biological Applications. Molecules.

[3] Saliyeva L.M., Dyachenko I.V., Danyliuk I.Y., Vovk M.V. (2022). Di-, tetra-, and perhydropyrrolo[1,2-a]imidazoles: The Methods of Synthesis and Some Aspects of Application. Chem. Heterocycl. Compd..

[4] Hyjek K., Jodłowski P. (2023). Metal-organic frameworks for efficient drug adsorption and delivery. Sci. Rad..

[5] Komkov A.V., Sukhanova A.А., Menchikov L.G., Zavarzin I.V. (2022). o-Aminopyrimidine Aldehydes and Ketones: Synthesis and use as Precursors to Fused Pyrimidines. Chem. Heterocycl. Compd..

[6] Danyliuk I.Y., Vovk M.V. (2022). Tetrahydroazepines with an annulated five-membered heteroaromatic ring. Chem. Heterocycl. Compd..

[7] Obernikhina N.V., Kachaeva M.V., Kachkovsky O.D., Brovarets V.S. (2022). In silico Study of Conjugated Nitrogen Heterocycles Affinity in their Biological Complexes. Chem. Heterocycl. Compd..

[8] Khomenko D.M., Doroshchuk R.O., Ohorodnik Y.M., Ivanova H.V., Zakharchenko B.V., Raspertova I.V., Vaschenko O.V., Dobrydnev A.V., Grygorenko O.O., Lampeka R.D. (2022). Expanding the chemical space of 3(5)-functionalized 1,2,4-triazoles. Chem. Heterocycl. Compd..

[9] Zhang S., Xu Z., Gao C., Ren Q.-C., Chang L., Lv Z.-S., Feng L.-S. (2017). Triazole derivatives and their anti-tubercular activity. Eur. J. Med. Chem..

[10] Keri R.S., Patil S.A., Budagumpi S., Nagaraja B.M. (2015). Triazole: A Promising Antitubercular Agent. Chem. Biol. Drug. Des..

[11] Chu X.M., Wang C., Wang W.L., Liang L.L., Liu W., Gong K.K., Sun K.L. (2019). Triazole derivatives and their antiplasmodial and antimalarial activities. Eur. J. Med. Chem..

[12] Xia Y., Liu Y., Wan J., Wang M., Rocchi P., Qu F., Iovanna J.L., Peng L. (2009). Novel Triazole Ribonucleoside Down-Regulates Heat Shock Protein 27 and Induces Potent Anticancer Activity on Drug-Resistant Pancreatic Cancer. J. Med. Chem..

[13] Groll A.H., Townsend R., Desai A., Azie N., Jones M., Engelhardt M., Schmitt-Hoffman A., Brüggemann R.J.M. (2017). Drug-drug interactions between triazole antifungal agents used to treat invasive aspergillosis and immunosuppressants metabolized by cytochrome P450 3A4. Transpl. Infect. Dis..

[14] Lass-Flörl C. (2011). Triazole Antifungal Agents in Invasive Fungal Infections. Drugs.

[15] Groll A.H., Townsend R., Desai A., Azie N., Jones M., Engelhardt M., Schmitt-Hoffman A., Brüggemann R.J.M. (2008). The Enzymatic Basis of Drug-Drug Interactions with Systemic Triazole Antifungals. Clin. Pharmacokinet..

[16] Ayati A., Emami S., Foroumadi A. (2016). The importance of triazole scaffold in the development of anticonvulsant agents. Eur. J. Med. Chem..

[17] Kharb R., Sharma P.C., Yar M.S. (2011). Pharmacological significance of triazole scaffold. J. Enzyme Inhib. Med. Chem..

[18] Kras J., Sadowski M., Zawadzińska K., Nagatsky R., Woliński P., Kula K., Łapczuk A. (2023). Thermal [3+2] cycloaddition reactions as most universal way for the effective preparation of five-membered nitrogen containing heterocycles. Sci. Rad..

[19] Tiwari G., Khanna A., Mishra V.K., Sagar R. (2023). Recent developments on microwave-assisted organic synthesis of nitrogen- and oxygen-containing preferred heterocyclic scaffolds. RSC Adv..

[20] Appukkuttan P., Mehta V.P., Van der Eycken E.V. (2009). Microwave-assisted cycloaddition reactions. Chem. Soc. Rev..

[21] Domingo L.R., Ríos-Gutiérrez M., Pérez P. (2023). Why is phenyl azide so unreactive in [3+2] cycloaddition reactions? Demystifying Sustmann’s paradigmatic parabola. Org. Chem. Front..

[22] Gomonov K.A., Pilipenko I.A. (2023). Formation of Five- and Six-membered Oxygen-containing Heterocycles on the Basis of 1-halo-1-nitroalkenes. Chem. Heterocycl. Compd..

[23] Milišiūnaitė V., Plytninkienė E., Bakšienė R., Bieliauskas A., Krikštolaitytė S., Račkauskienė G., Arbačiauskienė E., Šačkus A. (2021). Convenient Synthesis of Pyrazolo[4′,3′:5,6]pyrano[4,3-c][1,2]oxazoles via Intramolecular Nitrile Oxide Cycloaddition. Molecules.

[24] Zawadzińska K., Ríos-Gutiérrez M., Kula K., Woliński P., Mirosław B., Krawczyk T., Jasiński R. (2021). The Participation of 3,3,3-Trichloro-1-nitroprop-1-ene in the [3+2] Cycloaddition Reaction with Selected Nitrile N-Oxides in the Light of the Experimental and MEDT Quantum Chemical Study. Molecules.

[25] Sadowski S., Mudyna A., Knap K., Demchuk O.M., Łapczuk A. (2023). Synthesis of (Z)-N-aryl-C-(pyrid-3-yl)-nitrones. Sci. Rad..

[26] Jasiński E., Żmigrodzka M., Dresler E., Kula K. (2017). A Full Regioselective and Stereoselective Synthesis of 4-Nitroisoxazolidines via Stepwise [3+2] Cycloaddition Reactions between (Z)-C-(9-Anthryl)-N-arylnitrones and (E)-3,3,3-Trichloro-1-nitroprop-1-ene: Comprehensive Experimental and Theoretical Study. J. Heterocycl. Chem..

[27] Woliński P., Kącka-Zych A., Dziuk B., Ejsmont K., Łapczuk-Krygier A., Dresler E. (2019). The structural aspects of the transformation of 3-nitroisoxazoline-2-oxide to 1-aza-2,8-dioxabicyclo[3.3.0]octane derivatives: Experimental and MEDT theoretical study. J. Mol. Struct..

[28] Żmigrodzka M., Sadowski M., Kras J., Desler E., Demchuk O.M., Kula K. (2022). Polar [3+2] cycloaddition between N-methyl azomethine ylide and trans-3,3,3-trichloro-1-nitroprop-1-ene. Sci. Rad..

[29] Rostovtsev V.V., Green L.G., Fokin V.V., Sharpless K.B. (2002). A stepwise huisgen cycloaddition process: Copper(I)-catalyzed regioselective “ligation” of azides and terminal alkynes. Angew. Chem. Int. Ed. Engl..

[30] Levandowski B.J., Gamache R.F., Murphy J.M., Houk K.N. (2018). Readily Accessible Ambiphilic Cyclopentadienes for Bioorthogonal Labeling. J. Am. Chem. Soc..

[31] Yoshida S., Kuribara T., Ito H., Meguro T., Nishiyama Y., Karaki F., Hatakeyama Y., Koike Y., Kii I., Hosoya T. (2019). A facile preparation of functional cycloalkynes via an azide-to-cycloalkyne switching approach. Chem. Commun..

[32] Siadati S.A., Rezazadeh S. (2022). The extraordinary gravity of three atom 4π-components and 1,3-dienes to C20-nXn fullerenes; a new gate to the future of Nano technology. Sci. Rad..

[33] Boren B.C., Narayan S., Rasmussen L.K., Zhang L., Zhao H., Lin Z., Jia G., Fokin V.V. (2008). Ruthenium-Catalyzed Azide−Alkyne Cycloaddition: Scope and Mechanism. J. Am. Chem. Soc..

[34] Abu-Orabi S.T., Atfah M.A., Jibril I., Mari’i F.M., Ali A.A.-S. (1989). Dipolar cycloaddition reactions of organic azides with some acetylenic compounds. J. Heterocycl. Chem..

[35] Kula K., Kącka-Zych A., Łapczuk-Krygier A., Wzorek Z., Nowak A.K., Jasiński R. (2021). Experimental and Theoretical Mechanistic Study on the Thermal Decomposition of 3,3-diphenyl-4-(trichloromethyl)-5-nitropyrazoline. Molecules.

[36] Jasiński R. (2015). A stepwise, zwitterionic mechanism for the 1,3-dipolar cycloaddition between (Z)-C-4-methoxyphenyl-N-phenylnitrone and gem-chloronitroethene catalysed by 1-butyl-3-methylimidazolium ionic liquid cations. Tetrahedron Lett..

[37] Jasiński R. (2020). A new insight on the molecular mechanism of the reaction between (Z)-C,N-diphenylnitrone and 1,2-bismethylene-3,3,4,4,5,5-hexamethylcyclopentane. J. Mol. Graph. Model..

[38] Domingo L.R., Saéz J.A., Zaragozá R.J., Arnó M. (2008). Understanding the Participation of Quadricyclane as Nucleophile in Polar [2σ + 2σ + 2π] Cycloadditions toward Electrophilic π Molecules. J. Org. Chem..

[39] Domingo L.R., Ríos-Gutiérrez M. (2023). A Useful Classification of Organic Reactions Based on the Flux of the Electron Density. Sci. Rad..

[40] Mondal A., Mohammad-Salim H.A., Acharjee N. (2023). Unveiling substituent effects in [3+2] cycloaddition reactions of benzonitrile N-oxide and benzylideneanilines from the molecular electron density theory perspective. Sci. Rad..

[41] Ríos-Gutiérrez M., Domingo L.R. (2019). Unravelling the Mysteries of the [3+2] Cycloaddition Reactions. Eur. J. Org. Chem..

[42] Domingo L.R., Ríos-Gutiérrez M., Pérez P. (2022). Unveiling the Chemistry of Higher-Order Cycloaddition Reactions within the Molecular Electron Density Theory. Chemistry.

[43] Domingo L.R., Aurell M.J., Pérez P., Contreras R. (2002). Quantitative characterization of the global electrophilicity power of common diene/dienophile pairs in Diels–Alder reactions. Tetrahedron.

[44] Domingo L.R., Ríos-Gutiérrez M., Pérez P. (2016). Applications of the Conceptual Density Functional Theory Indices to Organic Chemistry Reactivity. Molecules.

[45] Domingo L.R. (2016). Molecular Electron Density Theory: A Modern View of Reactivity in Organic Chemistry. Molecules.

[46] Tolstikov G.A., Shults E.E., Malikova T.S., Spirikhin L.V. (1994). The Importance of Preliminary Orientation in [4+2]-Cycloadditions of Dienes and Dienophiles with Complex Structures. Mendeleev Commun..

[47] Aitouna A.O., Barhoumi A., Zeroual A. (2023). A Mechanism Study and an Investigation of the Reason for the Stereoselectivity in the [4+2] Cycloaddition Reaction between Cyclopentadiene and Gem-substituted Ethylene Electrophiles. Sci. Rad..

[48] Mondal A., Acharjee N. (2023). Unveiling the exclusive stereo and site selectivity in [3+2] cycloaddition reactions of a tricyclic strained alkene with nitrile oxides from the molecular electron density theory perspective. Chem. Heterocycl. Comp..

[49] Kula K., Sadowski M. (2023). Regio- and stereoselectivity of [3+2] cycloaddition reactions between (Z)-1-(anthracen-9-yl)-N-methyl nitrone and analogs of trans-β-nitrostyrene on the basis of MEDT computational study. Chem. Heterocycl. Comp..

[50] Gillies C.W., Gillies J.Z., Suenram D.R., Lovas F.J., Kraka E., Cremer D. (1991). Van der Waals complexes in 1,3-dipolar cycloaddition reactions: Ozone-ethylene. J. Am. Chem. Soc..

[51] Gillies J.Z., Gillies C.W., Lovas F.J., Matsumura K., Suenram R.D., Kraka E., Cremer D. (1991). Van der Waals complexes of chemically reactive gases: Ozone-acetylene. J. Am. Chem. Soc..

[52] Lupinski J.H. (1963). The chargé transfer complex between β-carotene and iodine. II. Characterization of complex. J. Phys. Chem..

[53] Bahnick D.A., Person W.B. (1968). Raman Intensity Study of Charge-Transfer Complexes of ICN. J. Chem. Phys..

[54] Domingo L.R. (2014). A new C–C bond formation model based on the quantum chemical topology of electron density. RSC Adv..

[55] Woliński P., Kącka-Zych A., Wróblewska A., Wielgus E., Dolot R., Jasiński R. (2023). Fully Selective Synthesis of Spirocyclic-1,2-oxazine N-Oxides via Non-Catalysed Hetero Diels-Alder Reactions with the Participation of Cyanofunctionalysed Conjugated Nitroalkenes. Molecules.

[56] Kula K., Łapczuk A., Sadowski M., Kras J., Zawadzińska K., Demchuk O.M., Gaurav G.K., Wróblewska A., Jasiński R. (2022). On the Question of the Formation of Nitro-Functionalized 2,4-Pyrazole Analogs on the Basis of Nitrylimine Molecular Systems and 3,3,3-Trichloro-1-Nitroprop-1-Ene. Molecules.

[57] Woliński P., Kącka-Zych A., Demchuk O.M., Łapczuk-Krygier A., Mirosław B., Jasiński R. (2020). Clean and molecularly programmable protocol for preparation of bis-heterobiarylic systems via a domino pseudocyclic reaction as a valuable alternative for TM-catalyzed cross-couplings. J. Clean. Prod..

[58] Jasiński R. (2018). β-Trifluoromethylated nitroethenes in Diels-Alder reaction with cyclopentadiene: A DFT computational study. J. Fluor. Chem..

[59] Jasiński R. (2014). Searching for zwitterionic intermediates in Hetero Diels–Alder reactions between methyl α,p-dinitrocinnamate and vinyl-alkyl ethers. Comput. Theor. Chem..

[60] Krokidis K., Noury S., Silvi B. (1997). Characterization of Elementary Chemical Processes by Catastrophe Theory. J. Phys. Chem. A..

[61] Frisch M.J., Trucks G.W., Schlegel H.B., Scuseria G.E., Robb M.A., Cheeseman J.R., Scalmani G., Barone V., Mennucci B., Petersson G.A. (2009). Gaussian 09.

[62] Kącka-Zych A. (2021). The Molecular Mechanism of the Formation of Four-Membered Cyclic Nitronates and Their Retro (3 + 2) Cycloaddition: A DFT Mechanistic Study. Molecules.

[63] Fryźlewicz A., Kącka-Zych A., Demchuk O.M., Mirosław B., Woliński P., Jasiński R. (2021). Green synthesis of nitrocyclopropane-type precursors of inhibitors for the maturation of fruits and vegetables via domino reactions of diazoalkanes with 2-nitroprop-1-ene. J. Clean. Prod..

[64] Pérez P., Domingo L.R., Aizman A., Contreras R. (2008). The electrophilicity index in organic chemistry. Theor. Aspects Chem. React..

[65] Perez P., Domingo L.R., Aurell M.J., Contreras R. (2003). Quantitative characterization of the global electrophilicity patternof some reagents involved in 1,3-dipolar cycloaddition reactions. Tetrahedron.

[66] Parr R.G., Szentpály L.V., Liu S. (1999). Electrophilicity Index. J. Am. Chem. Soc..

[67] Domingo L.R., Chamorro E., Pérez P. (2008). Understanding the Reactivity of Captodative Ethylenes in Polar Cycloaddition Reactions. A Theoretical Study. J. Org. Chem..

[68] Domingo L.R., Pérez P., Sáez J.A. (2013). Understanding the local reactivity in polar organic reactions through electrophilic and nucleophilic Parr functions. RSC Adv..

[69] Cossi M., Rega N., Scalmani G., Barone V. (2003). Energies, Structures, and Electronic Properties of Molecules in Solution with the C-PCM Solvation Model. J. Comp. Chem..

[70] Kącka-Zych A. (2021). Understanding the uniqueness of the stepwise [4 + 1] cycloaddition reaction between conjugated nitroalkenes and electrophilic carbene systems with a molecular electron density theory perspective. Int. J. Quant. Chem..

[71] Jasiński R. (2022). Stepwise, zwitterionic course of hetero-Diels–Alder reaction between 1,2,4-triazine molecular systems and 2-cyclopropylidene-1,3-dimethylimidazoline. Chem. Heterocycl. Comp..

[72] Becke A.D., Edgecombe K.E. (1990). A simple measure of electron localization in atomic and molecular systems. J. Chem. Phys..

[73] Noury S., Krokidis X., Fuster F., Silvi B. (1999). Computational Tools for the Electron Localization Function Topological Analysis. Comput. Chem..

[74] Dennington R., Keith T.A., Millam J.M. (2016). GaussView Version 6.

